# Classification of Standing and Walking States Using Ground Reaction Forces

**DOI:** 10.3390/s21062145

**Published:** 2021-03-18

**Authors:** Ji Su Park, Sang-Mo Koo, Choong Hyun Kim

**Affiliations:** 1 Center for Bionics, Korea Institute of Science and Technology, Seoul 02792, Korea; gene0219@kist.re.kr; 2 Electronic Materials Engineering, Kwangwoon University, Seoul 01890, Korea

**Keywords:** gait analysis, wearable devices, insole, force sensing resistors, ground reaction force, center of pressure, waveform length, m-health, activities of daily living

## Abstract

The operation of wearable robots, such as gait rehabilitation robots, requires real-time classification of the standing or walking state of the wearer. This report explains a technique that measures the ground reaction force (GRF) using an insole device equipped with force sensing resistors, and detects whether the insole wearer is standing or walking based on the measured results. The technique developed in the present study uses the waveform length that represents the sum of the changes in the center of pressure within an arbitrary time window as the determining factor, and applies this factor to a conventional threshold method and an artificial neural network (ANN) model for classification of the standing and walking states. The results showed that applying the newly developed technique could significantly reduce classification errors due to shuffling movements of the patient, typically noticed in the conventional threshold method using GRF, i.e., real-time classification of the standing and walking states is possible in the ANN model. The insole device used in the present study can be applied not only to gait analysis systems used in wearable robot operations, but also as a device for remotely monitoring the activities of daily living of the wearer.

## 1. Introduction

Walking is necessary for performing most of our daily activities. However, gait disturbances may occur due to neurological disorders, such as spinal cord disorder, stroke, and Parkinson’s disease, or accidents such as a fall. Gait disturbance can cause significant discomfort in performing the activities of daily living (ADL); thus, gait rehabilitation is absolutely necessary to improve the quality of life of patients suffering from gait disturbance [1,2]. Conventional gait rehabilitation methods are based on the concept of simple and repetitive physical therapy assisted by rehabilitation therapists, and consequently, the treatment outcome may vary depending on the skill and experience of the therapist. Accordingly, studies have been conducted to operate gait rehabilitation robots using actuators and determine the motion intention by receiving feedback from the bio-signals of the patients to achieve more effective and quantifiable gait rehabilitation effect.

To ensure the effective operation of gait rehabilitation robots, it is essential to apply an algorithm that can detect the gait phase of the wearer [3,4]. Various methods have been developed for gait phase detection, including applying a heuristic threshold algorithm using empirically set thresholds or machine learning for detecting the gait phase after acquiring various bio-signals by using the ground reaction force (GRF), accelerometer (Acc), and gyros*COP*e (Gyro) [5,6,7,8].

Algorithms that detect the gait phases using GRF often use the method of detecting the gait phase transition when the GRF measured at the heel or metatarsal bone exceeds a certain threshold. Mariani et al. [6] set the GRF threshold based on the body weight of the insole wearer, whereas Catalfamo et al. [7] set the threshold using the ratio between the maximum and minimum GRF values. Moreover, Yu et al. [8] classified the gait phase using the ratio between the GRF values measured at the left and right feet. In a previous study, the authors of the present study had developed a technique for real-time classification of gait into eight phases by using an insole device equipped with force sensing resistors (FSRs), which were developed to measure the GRF and examine the changes in the center of pressure (*COP*) [9].

The method using threshold values has also been applied to algorithms using the inertial measurement unit (IMU) data. Kotiadis et al. [10] proposed an algorithm that uses an Acc to classify the gait phase of patients with foot drop, whereas Mannini et al. [11] processed the Acc data with the Hidden Markov Model to classify the gait into four phases. Selles et al. [12] used the Acc placed on the calf to analyze the gait phase of transtibial amputee patients, demonstrating that the Acc could be used to classify the phases of abnormal gait. The use of the Acc or Gyro data, as described above, enables the observation of the movement of the swing foot. However, according to a study by Willemsen et al. [13], using an inertial sensor in gait analysis is known to show a delay of 20–40 ms, when compared with the use of a GRF. Moreover, because an inertial sensor is operated based on the continuous integration of data over time, a drift phenomenon occurs, caused by the accumulation of measurement errors over time.

On the other hand, the method of measuring the timing of heel contact using GRF for classification of the gait phase is considered the “gold standard” for gait phase analysis techniques [14], while also offering various advantages, including: (1) less deviation in the measurement signals according to sensor placement; (2) no data drift found when using the inertial sensor; (3) insole devices used to measure the GRF can be worn continuously during ADL; and (4) real-time applicability owing to short data computation time [6,7,8]. However, because the GRF cannot be measured accurately in patients with foot drop owing to shuffling, the accuracy of classifying the gait phases has been determined to be poorer than that achieved when using an Acc [15,16]. To overcome such disadvantages, various studies have attempted to enhance the accuracy of gait phase classification by simultaneously using multiple sensors to minimize the effect of shuffling. However, such efforts enhanced the complexity of the device or system, resulting in reduced usability [16,17,18,19].

A study by Bar-Haim et al. [20] converted the *COP* information calculated from the GRF data into entropy to assess the level of rehabilitation in patients. It was determined that although the GRF was an appropriate factor for assessing the gait complexity (or dynamic stability) in such a case, it was not an appropriate factor for gait phase classification.

The operation of wearable robots, such as gait rehabilitation robots, requires the real-time classification of the standing or walking state of the wearer. To develop a technique for classification of the standing and walking states, hereinafter called state classification, the authors conducted the study with the following objectives: (1) develop the technique for distinguishing between two different states, namely, the standing state where the person is not moving, and the walking state where the person is moving; (2) use GRF data as the data for state classification; and (3) develop a technique to significantly reduce the measurement errors caused by shuffling. Accordingly, the most appropriate factors for state classification were derived and selected from the GRF data in the study, and the state classification accuracy was examined by applying a machine learning method and threshold method that used the threshold values of these factors.

There is no precedent case of applying the neural network technique to a study that distinguishes between standing and states. This study will remarkably improve the accuracy of distinguishing standing and walking states by applying a neural network technique.

## 2. Methods

### 2.1. Hardware Description

**Figure 1:**
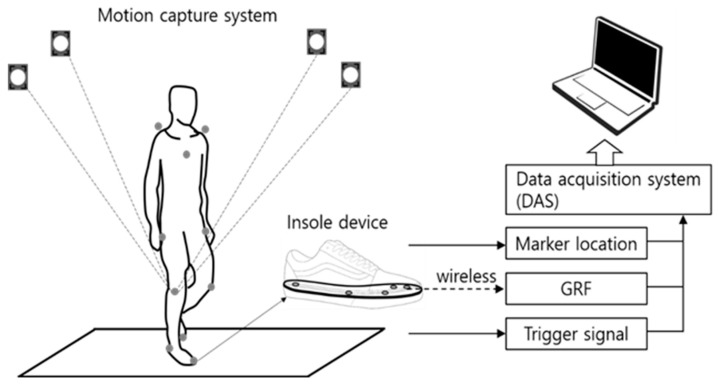
Experimental setup.

In the gait experiments, an insole-type GRF measurement device and a motion capture system (Osprey, Motion Analysis Corporation, CA 95403, USA) were used simultaneously, as shown in Figure 1. The participants wore tight-fitting experimental garment (top and bottom) to which motion capture reflective markers were attached. The reflective markers were attached to positions defined by the Helen Hayes Model [21]. Subsequently, the participants wore an insole device matching their foot size for the GRF measurement.

The GRF measurement device was developed by the authors of the present study. It consisted of an insole equipped with FSRs (FSR 402, Interlink Electronics, Inc. CA 93012, USA), which was inserted into the shoe [9]. Details of the insole device are explained in Appendix A. The GRF and motion capture data were collected using a data acquisition system (NI 6259, National Instruments, TX 78759-3504, USA). Because the GRF measurement device and motion capture system used in the study have different data sampling rates (100 Hz and 30 Hz, respectively), data synchronization was needed. Accordingly, the experiment operator pressed a synchronization signal generation button at the start and end of each experiment to generate a constant voltage trigger signal of 5 V and 0 V, respectively. The trigger signals were saved along with the GRF data and positional data of the motion capture markers to establish the reference points for synchronization. Upon completion of the experiment, the data at the starting and ending points of the experiment, as set by the trigger signals, were interpolated to obtain uniform numbers of data in the same time interval. The acquired GRF data were used for state classification, and the motion capture marker position data were used as the reference data for the actual state classification. Python was used for post-processing of the data acquired by the data acquisition system (DAS).

### 2.2. Participants

The study population for the gait experiments included 32 participants: 28 healthy adults and 4 patients with stroke-induced hemiplegia to evaluate the effect of shuffling on the classification accuracy of standing and walking states (Table 1). The healthy adult group comprised 19 males (age 25 ± 3 years, height 1.75 ± 5.82 m, weight 68 ± 7 kg) and 9 females (age 21 ± 1 years, height 1.61 ± 4.22 m, weight 51 ± 4 kg), who were capable of normal ambulation and had no trauma or neurological disorder.

The hemiplegia group comprised 4 males belonging to the functional ambulation category (FAC) level 5, who were capable of independent ambulation of 10 m without an assistive device.

### 2.3. Test Method

The participants in the gait experiment remained in the standing position while facing forward, and when prompted to start walking by the operator, they walked a distance of 5 m on a flat ground and stopped at the marked position. The participants were allowed to arbitrarily select how long they would stay in the standing state and their walking speed. Each participant repeated the gait experiment a total of 10 times.

The experimental protocol was approved by the Institutional Review Board (IRB) at the Korea Institute of Science and Technology (KIST). All participants provided written informed consent for the study prior to participation.

### 2.4. Selection and Evaluation of Factors

#### 2.4.1. Candidate Factors to Overcome Errors Caused by Foot Drop

**Figure 2:**
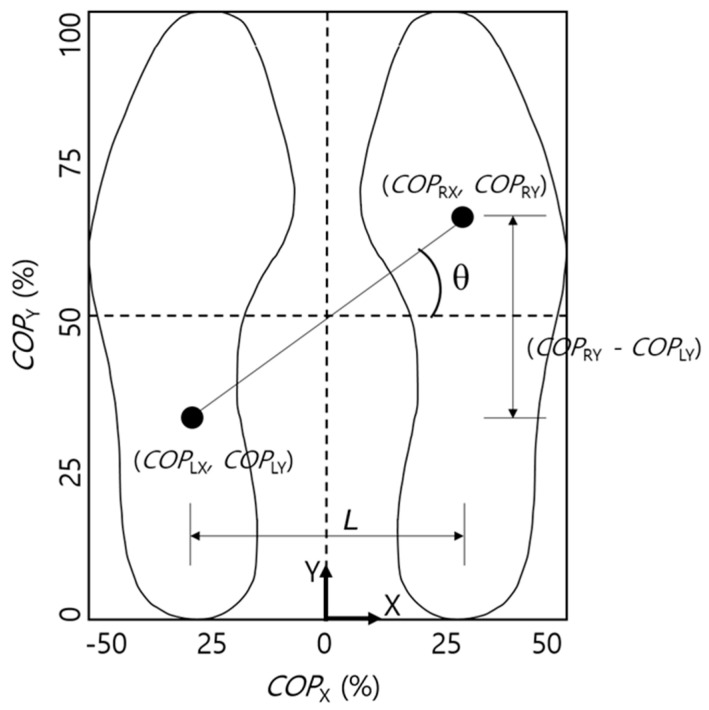
Schematic of center of pressure (*COP*) gradient.

When shuffling occurs due to foot drop, the magnitude of the GRF measured by an individual FSR is greatly affected, but the effect on the *COP* is relatively less because the *COP* is calculated using the GRF data measured by multiple FSRs. Accordingly, the present study aimed to use the *COP* for state classification. As shown in Figure 2, the GRF data were used to calculate the positions of the *COP* as follows.

(1) $$COP_{X}=\sum^{\;}FSR\;\cdot \;SP_{X}/\sum^{\;}FSR$$

(2) $$COP_{Y}=\sum^{\;}FSR\;\cdot \;SP_{Y}/\sum^{\;}FSR$$

(3) $$COP_{LX}=\sum^{\;}FSR_{L}\;\cdot \;SP_{LX}/\sum^{\;}FSR_{L}$$

(4) $$COP_{RX}=\sum^{\;}FSR_{R}\;\cdot \;SP_{RX}/\sum^{\;}FSR_{R}$$

(5) $$COP_{LY}=\sum^{\;}FSR_{L}\;\cdot \;SP_{LY}/\sum^{\;}FSR_{L}$$

(6) $$COP_{RY}=\sum^{\;}FSR_{R}\;\cdot \;SP_{RY}/\sum^{\;}FSR_{R}$$

Here, $COP_{X}$ and $COP_{Y}$ denote the positions of the combined *COP* in the x- and y-axis directions, respectively; $COP_{LX}$ and $COP_{RX}$ denote the positions of the *COP* in the x-axis direction, measured from the left and right foot, respectively; and $COP_{LY}$ and $COP_{RY}$ represent the positions of the *COP* in the y-axis direction, measured from the left and right foot, respectively. Regarding the sensor position (SP), the values defined by the authors in the previous study were used as the positional coordinates of the FSR [9]. With respect to the coordinate axes, the x- and y-axis were defined as the left/right and forward/backward directions when the body was facing forward, respectively.

As shown in Figure 2, as the participants began walking, the positions of the *COPs* of the left and right feet, projected on the surface, shifted by repeatedly alternating between the forward/backward directions. The angle (θ) was formed by the segment connecting these two points and the frontal plane, and $COP_{gradient}$ was calculated using the equation below. In this equation, L, which was defined as the distance between the left and right feet, was assumed to be the same as the width of the hips (Figure 2), and its value was set based on the height of the participant [22].

(7) $$\theta =COP_{gradient}=\arctan\left(\left(COP_{RY}-COP_{LY}\right)/L\right)\;(-\frac{\pi }{2}<COP_{gradient}\leq \frac{\pi }{2})$$

The amount of change in $COP_{gradient}$ over time was derived by dividing $COP_{gradient}$ by the sampling rate of the FSRs.

(8) $$\dot{COP}=\frac{d}{dt}COP_{gradient}$$

#### 2.4.2. Selection of Factor Based on Approximate Entropy

Entropy is reported to be a measure of the complexity of the deterministic dynamics of a time series [23]. In the field of gait analysis, the entropy of the *COP* value is used to test the therapeutic effect of rehabilitation or as an index for distinguishing between healthy adults and patients. Schmit et al. [24] compared the *COP* variations of patients with Parkinson’s disease against those of healthy elderly persons and determined that the former have relatively lower complexity in the *COP* variations. A study by Bar-Haim et al. [20] showed that the entropy of *COP* increased when the gait function of the patients was improved. Such results indicated that the pattern of change in the *COP* that appears during gait might be different between healthy adults and patients. Hence, applying the *COP* data from patients to an algorithm for gait detection developed using the *COP* data of healthy adults may not yield accurate detection results [15]. Therefore, unlike gait complexity assessment, it is necessary to identify the factors that do not show a significant difference between healthy adults and patients for state classification. 

**Figure 3:**
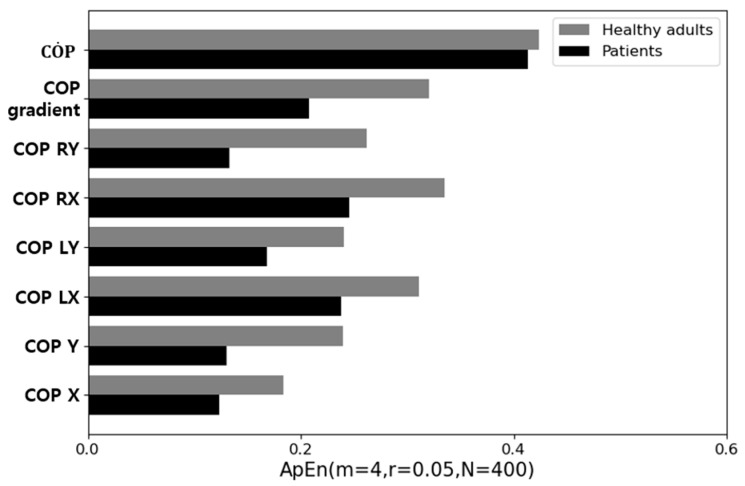
Comparison of approximate entropies of the factors.

$COP_{X}$, $COP_{Y}$, $COP_{LX}$, $COP_{RX}$, $COP_{LY}$, $COP_{RY}$, $COP_{gradient}$, and $\dot{COP}$ obtained from the GRF data were used to calculate the entropy values for healthy adult and patient groups; the results are shown in Figure 3. The approximate entropies (ApEn) of the variables listed above were calculated using the following steps. 

1. m number of sample data X(i), as defined by the pattern length, were generated.

(9) $$X\left(i\right)=\left[x_{i},\;x_{i+1},\;\cdots ,\;x_{i+m-1}\right]$$

2. The generated sample data were used to calculate their correlations by the following equation.

(10) $$C_{i}^{m}\left(r\right)=\frac{number\;of\;X\left(j\right)\;s.t.\;d\left[X\left(i\right),\;X\left(j\right)\right]\leq r}{N-m+1},\;d\left[X,\;X^{*}\right]=\underset{a}{\max}\left|u\left(a\right)-u^{*}\left(a\right)\right|$$

3. After determining the log of the correlation value, the mean log value was used to derive the cumulative entropy.

(11) $$\Phi^{m}\left(r\right)={\left(N-m+1\right)}^{-1}\sum_{i=1}^{N-m+1}\log\left(C_{i}^{m}\left(r\right)\right)$$

4. The cumulative entropy was used to calculate ApEn by the following equation.

(12) $$ApEn=\;\underset{N\to \infty }{\lim}\Phi^{m}\left(r\right)-\Phi^{m+1}\left(r\right)$$

In Figure 3, the factor that showed the smallest difference in entropy between the two groups was $\dot{COP}$, which represents the amount of change in $COP_{gradient}$. In other words, the results indicated that $\dot{COP}$ is the most appropriate factor for state classification. The explanation of the application of the entropy for $\dot{COP}$ is introduced in Appendix B.

#### 2.4.3. Waveform Length of $\dot{COP}$

$COP_{gradient}$ has a value of zero when the two feet alternate during gait or when standing with both feet parallel to each other. Therefore, the standing and walking states cannot be differentiated by calculating and comparing the $\dot{COP}$ values.

To rectify this issue, the waveform length was derived by combining $\dot{COP}$ within an arbitrary time window and using it for state classification, as shown below [25].

(13) $$COP_{W}=\sum_{i=a}^{window\;size}\dot{COP}$$

In other words, the gait data from the previous step are used when classifying the current walking state using $COP_{W}$.

### 2.5. Classification of Standing and Walking States

#### 2.5.1. Threshold Method

##### Timing Analysis Module (TAM) Method, Using the GRF Threshold

The Timing Analysis Module (TAM) method [7,8] used in previous studies was used to differentiate between the standing and walking states. This method determines the contact between the foot and ground based on the magnitude of the GRF, and the threshold is calculated using the following equation.

(14) $$GRF_{TH}=GRF_{min}+\left(GRF_{max}-GRF_{min}\right)\times \frac{10}{100}$$

Here, $GRF_{max}$ and $GRF_{min}$ represent the maximum and minimum values of the sum of GRFs measured by multiple FSRs, respectively. Because the *GRF* data were applied with the participants segregated into the healthy adult and patient groups, $GRF_{max}$ and $GRF_{min}$ were different for each group. The TAM method classifies the state as the standing state when the sum of the *GRFs* measured is greater than $GRF_{TH}$ from the above equation; otherwise, the state is classified as the walking state.

##### Using $COP_{w}$ Threshold

After setting the $COP_{W}$ threshold ($COP_{W.TH}$), the following criteria were applied to define the standing and walking states as 0 and 1, respectively.

(15) $$State=\;\{\begin{array}{c} 0,\;if\;COP_{w}<COP_{W.TH}\;\\ 1,\;if\;COP_{w}\geq COP_{W.TH} \end{array}$$

**Figure 4:**
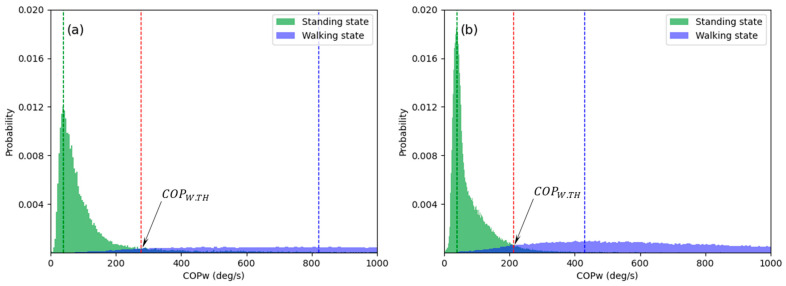
Histograms of the waveform length of $\dot{COP}$ ($COP_{W}$): (**a**) healthy adults (**b**) patients.

Figure 4 shows the probability of $COP_{W}$, which was calculated using the GRF data generated in the standing and walking states. In the histograms shown in Figure 4, $COP_{W.TH}$ was determined as $COP_{W}$ with the highest probability in the area of overlap of the standing and walking states. Therefore, the healthy adult and patient groups have different threshold values, as shown in Figure 4a,b. The state determined using Equation (15) was compared with the actual state determined by a motion capture system to assess the accuracy of the state classification technique proposed in the present study.

##### 2.5.2. Artificial Neural Network Model

**Figure 5:**
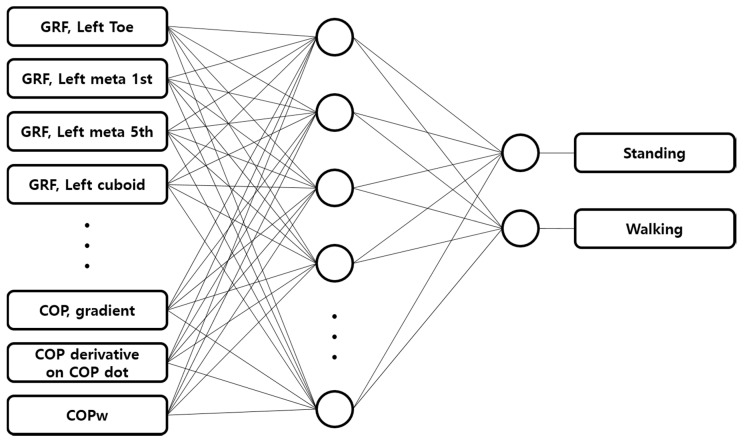
Diagram of the developed artificial neural network (ANN) model.

For comparison with the aforementioned threshold method, a machine learning-based state classification model was developed, as shown in Figure 5. In the ANN model, GRF, $COP_{X}$, $COP_{Y}$, $COP_{LX}$, $COP_{RX}$, $COP_{LY}$, $COP_{RY}$, $COP_{gradient}$, $\dot{COP}$, and $COP_{W}$ were used as the input data, while supervised learning was performed by applying the state classification results from the motion capture system as the learning data. The learning data were normalized by dividing by the maximum value that appeared for each type of input data to reduce the influence due to the size of each data value. The ANN model was developed as a single layer to allow the use of Garson’s algorithm for assessing the relative importance of the factors, and it consisted of 20 nodes. In the model for state classification of the healthy adult group, 200 sets of experimental data randomly selected from a total 280 sets of gait experiment data were used for model learning, and the remaining 80 sets of experimental data were used for state classification. In the model for state classification of the patient group, 30 sets of experimental data randomly selected from 40 sets of gait experiment data were used for model learning, and the remaining 10 sets of experimental data were used for state classification.

Moreover, Garson’s algorithm was applied to the factors used in the ANN model to assess the relative importance of each factor [26,27]. The relative importance of each input factor used in the model created after the completion of learning was calculated by the following equation. Relative importance represents a relative value and the sum of the relative importance of all input factors used in a single system should be 100%.

(16) $$\mathrm{Relative}\;\mathrm{Importance}\;\left(\%\right)=\;\frac{\sum^{\;}\frac{\left|w_{in}\right|\left|w_{out}\right|}{\left|w_{out}\right|}}{\sum^{\;}\sum^{\;}\frac{\left|w_{in}\right|\left|w_{out}\right|}{\left|w_{out}\right|}}$$

## 3. Results

According to a study by Pappas et al. [15], gait analysis algorithms for treatment or rehabilitation, which show a classification accuracy of <90%, are difficult to use in actual clinical practice. Accordingly, the present study set the goal of achieving a classification accuracy of ≥90% for the state classification algorithm developed in the present study.

### 3.1. State Classification Accuracy When Using Threshold Methods

**Figure 6:**
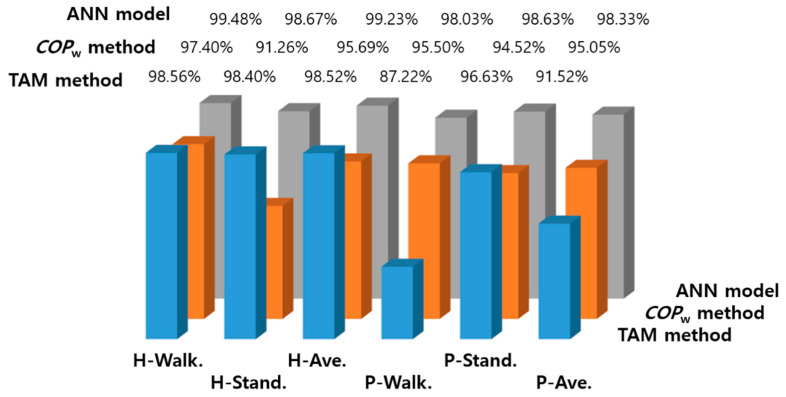
Comparison of the classification accuracies: H-, healthy adults; P-, patients.

Figure 6 shows the results of the state classification accuracy obtained in the present study. The mean state classification accuracies in the gait experiments on the healthy adult group were 98.52% and 95.69% when using the TAM method and threshold method using $COP_{W}$, respectively, showing that both methods exceeded the target classification accuracy of ≥90%. In the healthy adult group, the classification accuracies for the standing and walking states were higher when the TAM method was used, when compared with the threshold method using $COP_{W}$. This could be attributed to the fact that the threshold method using $COP_{W}$ is influenced by the previously collected data when calculating the waveform length within an arbitrary time window. Therefore, such methods may not only incorrectly classify the current walking state, but also show classification delay.

The mean state classification accuracies in the gait experiments on the patient group were 91.52% and 95.05% when using the TAM method and $COP_{W}$, respectively, showing that both methods exceeded the target classification accuracy of ≥90%. However, the classification accuracy for the walking state (P-walk. in Figure 6) obtained using the TAM method was unsatisfactory at 87.22%. This may be attributed to the misinterpretation of the walking state as standing state due to shuffling by the patients when the GRF was measured in the swing foot.

**Figure 7:**
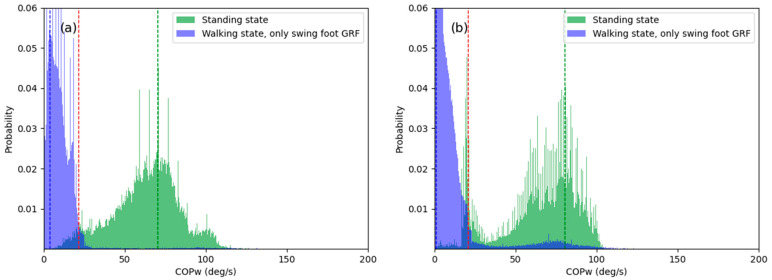
Histogram of ground reaction force (GRF) data: (**a**) healthy adults (**b**) patients.

Figure 7 shows the GRF data collected from gait experiments on the healthy adult and patient groups. The graphs show the sum of the GRFs of both feet in the standing state and the GRF of the swing foot in the walking state. In Figure 7a, which shows the GRF data for the healthy adult group, the mode value of the GRFs for the standing and walking states are separated from each other, whereas in Figure 7b, which shows the GRF data for the patient group, some of the GRF values measured at the swing foot overlaps with the mode value region of the GRF in the standing state. This indicated that the GRF was measured when shuffling occurred in the swing foot; consequently, an error occurred where the standing state was detected despite the fact that the patient was actually walking, which caused the state classification accuracy to decrease.

On the other hand, the classification accuracy of the threshold method using $COP_{W}$ for the walking state of the patient group (P-walk. in Figure 6) was high, reaching up to 95.50%. It is believed that such results were due to significant reduction in errors caused by shuffling by applying the *COP*-based factors, as intended by the authors.

In Table 2, the percentile of the GRF mode values in the walking state, measured by the TAM method, were 96.00% and 89.50% for the healthy adult and patient groups, respectively. Both groups also showed similar state classification accuracies of 98.56% and 87.22%, respectively. Moreover, the percentile of the GRF mode values in the standing state, measured by the threshold method using $COP_{W}$, were 90.00% and 94.50% for the healthy adult and patient groups, respectively; both groups also showed very similar state classification accuracies of 91.26% and 94.52%, respectively. Such results indicate that it is easier to classify between the standing and walking states when there is a greater separation between the datasets generated in those states. Therefore, it is inferred that using $COP_{W}$ as the factor for classifying the states in the patient group will be more effective than using GRF with the threshold method.

Overall, relatively higher classification accuracies were achieved by the TAM method using GRF for the healthy adult group (98.52%) and the threshold method using $COP_{W}$ for the patient group (95.05%). Moreover, applying the threshold method using $COP_{W}$ showed very high mean state classification accuracies of 95.69% and 95.05% for the healthy adult and patient groups, respectively, which suggests its applicability to actual clinical experiments. 

**Figure 8:**
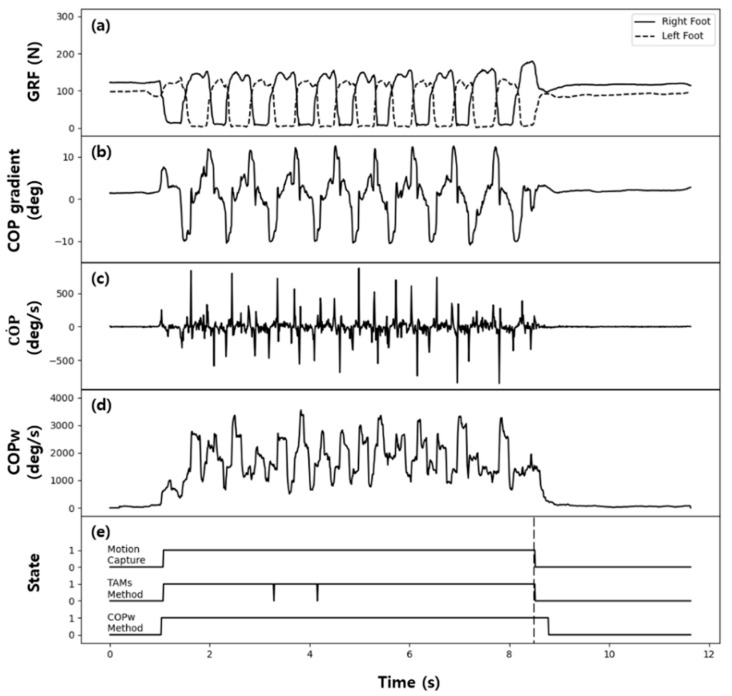
Typical test results for a healthy adult: (**a**) GRF, (**b**) $COP_{gradient}$, (**c**) $\;\dot{COP}$, (**d**) $\;COP_{W}$, (**e**) classification results.

**Figure 9:**
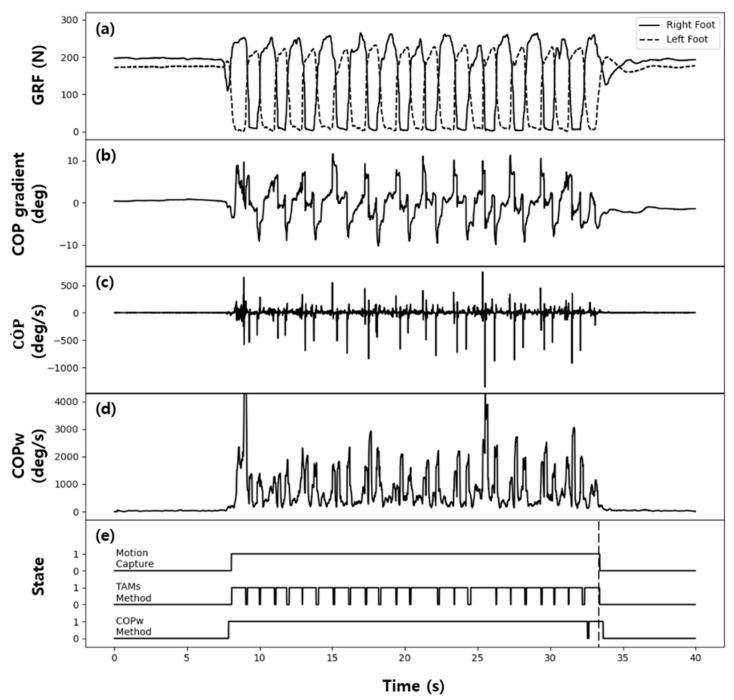
Typical test results for a patient: (**a**) GRF, (**b**) $COP_{gradient}$, (**c**) $\;\dot{COP}$, (**d**) $\;COP_{W}$, (**e**) classification results.

Figure 8 and Figure 9 show the GRF data measured in the gait experiments on the healthy adult and patient groups, respectively, and $\dot{COP}$ and $COP_{W}$ calculated from these data, along with the examples of state classification by the two methods—the TAM method and threshold method using $COP_{W}$. Figure 8e and Figure 9e show the actual state classification results (actual states) of the motion capture system and those of the TAM method and threshold method using $COP_{W}$ together for comparison. In Figure 8e, which shows the gait experiment results for the healthy adult group, both the TAM method and threshold method using $COP_{W}$ showed very similar results as the actual state classified by the motion capture system. The TAM method accurately classified the start and end of walking, with occasional error in misinterpreting the walking state as the standing state. The threshold method using $COP_{W}$ showed superior state classification accuracy than the TAM method, but it also showed delay in classifying the transition from the walking state to the standing state. This classification delay was due to using the data from the previous gait step when calculating $COP_{W}$, as described earlier, and additional time was required to avoid this influence.

In Figure 9e, which shows the gait experiment results for the patient group, several classification errors occurred when the TAM method was used for state classification. This classification error was due to the patient group showing shuffling of the swing foot during walking, which caused the walking state to be misinterpreted as the standing state. On the other hand, the threshold method using $COP_{W}$ showed a sharp decrease in the frequency of state classification errors and significant improvement in the classification accuracy, even in the patient group.

### 3.2. State Classification Accuracy by Machine Learning

As shown in Figure 6, the ANN model demonstrated very high mean state classification accuracies of 99.23% and 98.33% for the healthy adult and patient groups, respectively. In comparison with the threshold method using $COP_{W}$, the classification accuracy increased by 3.54% and 3.28% for the healthy adult and patient groups, respectively, and there was no noticeable classification delay.

**Figure 10:**
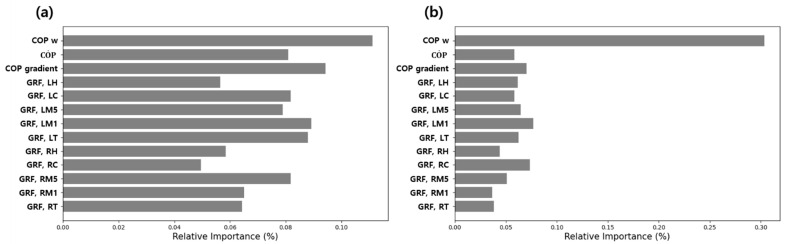
Relative importance of the factors: (**a**) healthy adult group (**b**) patient group.

Figure 10 shows the results of the relative importance of the input factors used in the ANN model, calculated using Equation (16). As shown in Figure 10, the factor with the highest relative importance in both the healthy adult and patient groups was $COP_{W}$. All other factors showed a relative importance within the range of 0.045–0.09 in both groups, whereas $COP_{W}$ showed a relative importance of 0.11 and 0.30 in the healthy adult and patient groups, respectively, which confirmed that it was the most important factor in both groups. Especially in the state classification model for the patient group, the relative importance of $COP_{W}$ was much greater than that of all other factors. Based on these results, it was determined that using $COP_{W}$ as the factor for machine learning-based state classification was the right decision.

## 4. Discussion

A recent study by Tang et al. [5] used three different threshold values for distinguishing between the standing and walking states, and proposed a method of tuning the threshold values at each step by using the maximum and minimum GRF values from the previous step, which they referred to as the self-tuning triple threshold algorithm (STTTA). However, STTTA had the inherent limitation of decreased state classification accuracy due to shuffling of the swing foot in the patient group. Djamaa et al. [28] used the GRF to classify the walking state as shuffle walk, toe walk, and normal walk, and demonstrated that such classification could be useful in diagnosing the presence of a disorder. However, this method too could not avoid the classification error caused by shuffling.

On the other hand, the application of $COP_{W}$, which was introduced as a new factor in the present study, to the threshold method yielded high mean state classification accuracies of 95.69% and 95.05% in the experiments on the healthy adult and patient groups, respectively, which indicated significant reduction in classification errors caused by shuffling.

**Figure 11:**
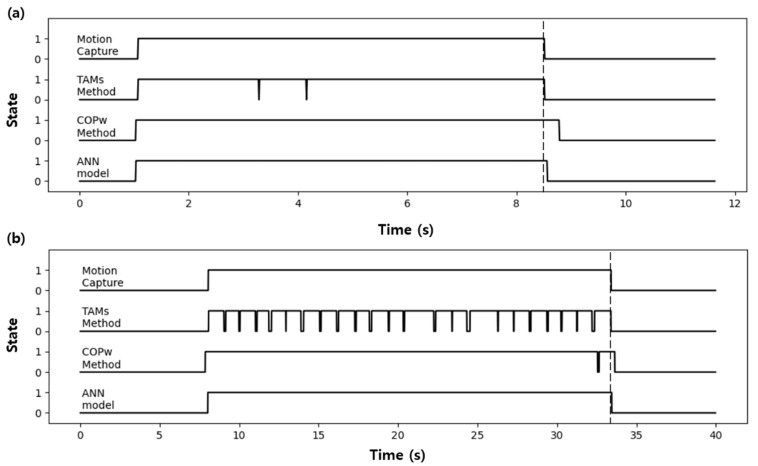
Comparison of the classification results using the test data: (**a**) healthy adults (**b**) patients.

Figure 11 shows the state classification by applying the experimental data shown in Figure 8 and Figure 9 to the ANN model. The results showed almost no state classification error. Moreover, the ANN model using $COP_{W}$ as the factor showed no classification delay, unlike the threshold method using $COP_{W}$.

Table 3 shows the delay in the state classification time in all gait experiments. The start and end time points of walking represented the time points when the motion capture marker placed on the solar plexus moved in the direction of walking, and the time delay was defined as the difference in time between these time points, observed by the motion capture system, and the time points for completion of state classification by the algorithm developed in the present study.

The most prominent gait characteristics of the patient group were (1) the time that the foot stayed in contact with the ground was relatively longer than that in the healthy adult group, owing to shuffling of the affected foot; (2) when the affected foot was lifted, a significant weight shift occurred to the unaffected foot. Therefore, when the GRF is used for state classification in the patient group, the foot touches down on the ground after a time delay owing to shuffling of the affected foot. Consequently, there is a delay in detecting the start of walking; the mean classification delay was found to be 52.1 ms. At the end of walking, the swing foot touches down on the ground earlier due to shuffling, when compared with the healthy adult group. Consequently, the classification process prematurely assumes that the participant is in the standing state; the mean classification delay was found to be −283.4 ms. The state classification delays in the healthy adult group when classified by the GRF were −11.8 ms and −2.6 ms at the start and end of walking, respectively. Because these classification delays were negligible, the results could be considered as real-time classification.

The threshold method using $COP_{W}$ showed the mean state classification delay at end of walking as 193.3 ms and 139.3 ms in the healthy adult and patient groups, respectively. There were two causes for this classification delay. The first cause was the fact that the waveform length was derived by a method that uses the sum of data within an arbitrary time window. Therefore, it was influenced by the data from the previous time window. In such cases, the calculation included the data from the previous walking state, even though the participant had already stopped, and, thus, the results may have continued to show the participant in the walking state. The second cause is that even when the participant stops walking, changes in the *COP* may continue to occur owing to the shaking of the body until the participant comes to a complete stop. Such changes may be reflected in $COP_{W}$ and the results may continue to show the participant in the walking state.

The mean time delay at the start of walking was −24.8 ms and −155.5 ms in the healthy adult and patient groups, respectively, meaning that state classification occurred before the start of walking in both groups. In the patient group, when the affected foot was lifted, excessive weight shift occurred to the unaffected foot. This caused a sudden increase in the amount of change in the *COP* before the knee movement, which could have been classified as the walking state. In the healthy adult group, anticipatory postural adjustments (APAs) appear, which are slight movements to maintain the balance before the body moves [29]. In other words, when the participants in the healthy adult group began walking from the standing position, the *COP* that was positioned between the two feet entered an imbalance phase of moving toward the heel of the swing foot before walking began. In the healthy adult group, such changes in the *COP* influenced $COP_{W}$, whereby the start of walking was classified prematurely.

When the ANN model was used, the time delay in state classification showed an absolute value of <10 ms regardless of the group and type of walking state. Therefore, classification using the ANN model can be considered as real-time state classification. In Figure 6, the results of state classification using the ANN model showed very high mean classification accuracies of 99.23% and 98.33% in the healthy adult and patient groups, respectively. Therefore, the ANN model developed in the present study can be viewed as a technique capable of real-time state classification with very high state classification accuracy, suggesting its suitability for application in clinical practice.

The lower classification accuracy of the threshold method using $COP_{W}$ for the standing state in the healthy adult group when compared with the other cases, as shown in Figure 6, can be explained based on the occurrence of APAs. In other words, the *COP* changed as the body wavered in the standing state, which would show a very similar pattern as the change in *COP* due to APAs before the start of walking. Therefore, wavering of the body in the standing state may have been erroneously classified as the walking state.

The method using $COP_{W}$ and the TAM method both require boolean-type logical comparison calculations, whereas the ANN model goes through the matrix calculation, so the amount of calculation is relatively increased compared to the above-mentioned two methods. However, the amount of matrix computation performed by the ANN model used in this study can be implemented in real-time.

## 5. Conclusions

The present study proposed a method for using the GRF data for state classification, while also reducing the errors caused by shuffling. An appropriate input factor was selected for state classification, and this factor was applied to the threshold method and machine learning-based method to examine the state classification accuracy.

Consequently, $COP_{w}$, the waveform length derived from the sum of all $\dot{COP}$ within an arbitrary time window, was selected as the state classification factor. The threshold method using $COP_{w}$ showed the mean classification accuracy of ≥95% in state classification experiments on the healthy adult and patient groups. This method was found to show significant improvement in the state classification error caused by shuffling, especially in the experiments on the patient group. However, because the data from the previous step were used in the computation to obtain $COP_{w}$, a classification delay was also detected. Moreover, changes in the *COP* that appear in the normal standing state may influence $COP_{w}$, which can lead to classification error.

However, the ANN model that used $COP_{w}$ as the factor showed excellent state classification accuracy of ≥98% in both the healthy adult and patient groups, while also showing no classification delay, which was observed in the threshold method using $COP_{w}$.

In conclusion, it was determined that the selection of $COP_{w}$ as the factor for state classification was an appropriate choice. Moreover, the ANN model using $COP_{w}$ cannot only fundamentally resolve the problem of state classification error caused by shuffling, but is also capable of real-time state classification. Furthermore, the GRF measurement device used in the present study was fabricated as an insole type that can be inserted into a shoe. Thus, it can be worn conveniently and operated for a long time, which enhances its applicability to actual clinical trials.

In future studies, the authors aim to develop a classification method with reduced state classification errors caused by differences in the gait characteristics between healthy adults and patients; change in direction of walking or turning during walking, and differences in the walking speed will be examined, so that the proposed method can be adopted in actual clinical experiments.

## Figures and Tables

**Table 1:** Participant information.

Condition	Normal, Male	Normal, Female	Patient
Number of persons	19	9	4
Age (years)	25 (S.D. 3)	21 (S.D. 1)	56 (S.D. 10)
Height (m)	1.75 (S.D. 0.06)	1.61 (S.D. 0.04)	1.67 (S.D. 0.05)
Weight (kg)	68 (S.D. 7)	51 (S.D. 4)	68 (S.D. 6)

**Table 2:** Classification accuracies of the threshold methods: TAM—Timing Analysis Module, $COP_{W}$ —the waveform length of $\dot{COP}$.

	TAM Method				$\mathrm{Method}\;\mathrm{Using}\;\;COP_{w}$			
	Healthy Adults		Patients		Healthy Adults		Patients	
	Standing	Walking	Standing	Walking	Standing	Walking	Standing	Walking
**Threshold value**	$GRF_{TH}=\;$21 (N)		20 (N)		$COP_{w.\mathrm{TH}}=\;$276 (deg/s)		212 (deg/s)	
**Mode value**	70 (N)	3 (N)	80 (N)	1 (N)	38 (deg/s)	820 (deg/s)	39 (deg/s)	430 (deg/s)
**Percentile**	3.73%	96.00%	9.00%	89.50%	90.00%	4.00%	94.50%	5.50%
**Classification accuracy**	98.40%	98.56%	96.63%	87.22%	91.26%	97.40%	94.52%	95.50%

**Table 3:** Variation of time delay in the gait experiments.

		Healthy Adults			Patients		
Walking Speed (m/s)		0.83 ± 0.08			0.29 ± 0.06		
Method		Threshold method		ANN	Threshold method		ANN
		GRF	$COP_{W}$		GRF	$COP_{W}$	
Time delay (ms)	at start of walking	−11.8 ± 7.8	−24.8 ± 21.7	3.2 ± 10.1	52.1 ± 26.1	−155.5 ± 97.2	−9.0 ± 13.9
	at stop of walking	−2.6 ± 12.4	193.3 ± 52.3	2.9 ± 9.3	−283.4 ± 104.7	139.3 ± 74.2	−3.9 ± 3.8

**Figure A1:**
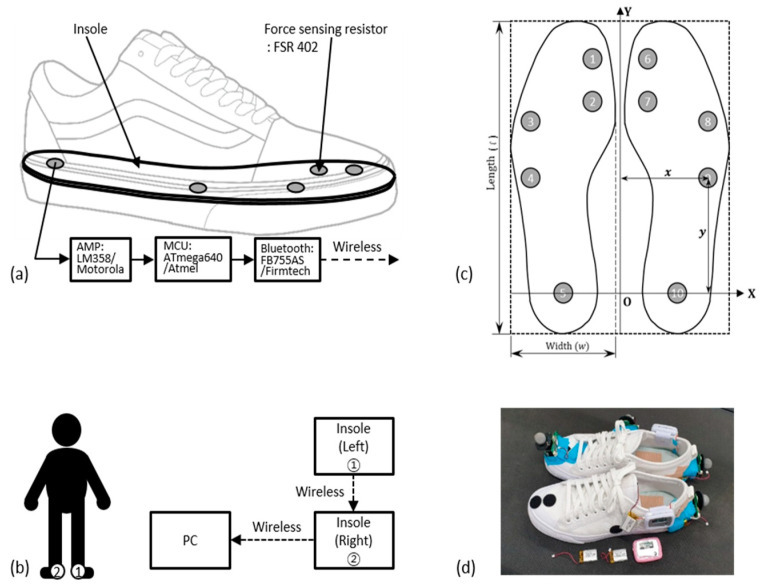
Gait analysis system: (**a**) insole device, (**b**) schematic of the data acquisition system (DAS), (**c**) location of force sensing resistors (FSRs) (**d**) developed system.

**Figure A2:**
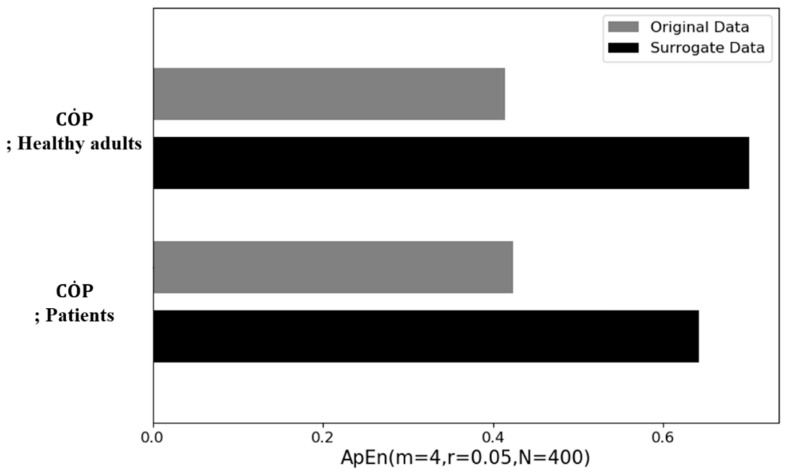
Comparison of approximate entropies of the $\dot{COP}$.

## Data Availability

The data presented in this study are available on request from the corresponding author. The data are not publicly available due to ethical concerns since they were obtained in a clinical trial.

## Appendix A

As illustrated in Figure A1a, the GRF measuring insole device developed in the present study comprised a total of 10 FSRs (FSR-402, Interlink Electronics, USA), of which five were attached to the bottom of the insole for each foot. The locations of the FSR attachment were as illustrated in Figure A1c, and as presented in Table A1. The locations were standardized to use the height of each participant [22]. The distance between the center points of both heels, depicted in Figure A1c, was assumed to be equivalent to the shoulder width.

The schematic of the gait analysis system is illustrated in Figure A1b. First, the GRF data collected from the left insole were transmitted to the right insole via a Bluetooth device; these data were subsequently combined with right foot GRF data and transmitted to a PC wirelessly by Bluetooth for use in the analysis.

**Table A1:** Location of FSRs for insole device.

No.	Position	**x**	**y**
1	Toe, Left	−0.191H ×0.5+0.25w	l ×0.85
2	1st Metatarsal, Left	−0.191H ×0.5+0.25w	l ×0.73
3	5th Metatarsal, Left	−0.191H ×0.5−0.25w	l ×0.64
4	Cuboid, Left	−0.191H ×0.5−0.25w	l ×0.50
5	Heel, Left	−0.191H ×0.5	l ×0.15
6	Toe, Right	0.191H ×0.5−0.25w	l ×0.85
7	1st Metatarsal, Right	0.191H ×0.5−0.25w	l ×0.73
8	5th Metatarsal, Right	0.191H ×0.5+0.25w	l ×0.64
9	Cuboid, Right	0.191H ×0.5+0.25w	l ×0.50
10	Heel, Right	0.191H ×0.5	l ×0.15
	*H=Height, l=0.152×H, w=0.055×H		

## Appendix B

In this study, the phase randomized Fourier surrogate method was used to determine whether $\dot{COP}$ was deterministic. In this method, the $\dot{COP}$ data is Fourier transformed, multiplied by a randomly assigned phase term, and then inverse Fourier transformed to obtain surrogate data [30,31]. ApEn was calculated for each of the original $\dot{COP}$ data and surrogate data, and if there was a difference between these two ApEn values, the original $\dot{COP}$ data was interpreted as a deterministic signal rather than a linear noise. The ApEn values calculated from the $\dot{COP}$ data of the healthy adult and patient groups obtained in this study are shown in Figure A2. It can be seen that both groups have different ApEn values obtained from original $\dot{COP}$ data and surrogate data, and these results indicate that the $\dot{COP}$ data obtained in this study is a deterministic signal.

.
